# Energy efficient trust aware secure routing algorithm with attribute based encryption for wireless sensor networks

**DOI:** 10.1038/s41598-025-03558-8

**Published:** 2025-06-05

**Authors:** M. Selvi, S. V. N. Santhosh Kumar, K. Thangaramya, H. Abdul Gaffar

**Affiliations:** 1 School of Computer Science and Engineering, Vellore Institute of Technology, Vellore, India; 2 School of Computer Science Engineering and Information Systems, Vellore Institute of Technology, Vellore, India

**Keywords:** Wireless sensor network, Security, Attribute based encryption, Trust-based secure routing protocol, Diffie–Hellman with RSA and clustering method, Energy science and technology, Electrical and electronic engineering

## Abstract

Attribute-Based Encryption (ABE) is one of the public-key cryptographic techniques which enforces a new method of security through effective control of access on the data which is stored or communicated in encrypted form using a variety of user defined access restrictions. ABE is implemented in a fully-fledged computer system, such as smartphones or embedded devices and it can be also used to enhance the security of network communication. In Attribute-Based Encryption, the encryption key will be associated with a set of attributes and these attributes are useful in the decryption process as well. Encrypting the data for numerous receivers can also be done with ABE, in such a way that only those with the right permissions can decrypt it later. The ABE provides a high level of security due to the wide range of key based qualities that can be defined, and it is also flexible enough to be used in a variety of communication and storage situations. In Wireless Sensor Networks (WSNs), the information is transmitted through wireless links from the sensors to a central location called the sink node. The nodes present in WSN are required to carry out the task of monitoring the environments, gather the necessary data through sensing, encrypt them and send them to the sink node. Therefore, security is a major concern for wireless communication since data is transmitted via an open wireless channel that can be accessed by malicious intruders. In order to handle the security problem, we propose a new Attribute-Based Encryption and Trust Based Secure Routing Algorithm (ABE-TBSRA) strategy by combining bilinear pairing and Diffie Hellman encryption scheme with RSA algorithm to encrypt and secure the data at the sender-side itself. In addition, we propose a new trust-based security model that uses three types of trusts namely direct, indirect, and historical trust values for improving the security. The major advantages of this proposed encryption and trust based secure routing algorithm include the enhancements in security level as well as packet delivery ratio, reliability of transaction and also the network throughput with reduced energy consumption and delay.

## Introduction

Recent developments in Wireless Sensor Network (WSN) technology have made it possible to create optimal cost and power efficient power efficient design of sensor nodes with multiple functions. In wireless sensor networks, a huge number of resource-limited sensor devices are dispersed throughout the deployment region. Communication and cryptosystems both rely heavily on the security and confidentiality of the information they exchange. Though many different security methods are available for securing WSN in the provision of reliable data delivery, cryptographic techniques are the most efficient techniques as they can ensure confidentiality and data integrity effectively. In cryptography, encrypted data can be deciphered by using crypt analysis techniques when the keys are not known. There are two main methods of encrypting data namely the encryption based on symmetric keys and asymmetric keys^1^. Symmetric key algorithms are easy to design and perform the implementation when it is compared to the public key algorithms due to the use of single key in this type of algorithm by the sender and the receiver. In public key algorithm, different keys are used by the sender and receiver and hence it is more complex with respect to design but provides enhanced security. Based on these requirements, we propose a new attribute-based encryption algorithm by extending the bilinear pairing and Diffie–Hellman encryption method with RSA algorithm for developing a new trust and encryption based secure routing protocol for secure delivery of data in WSNs.

In the past, many researchers developed encryption schemes for securing the data communicated through the network. Moreover, the Attribute Based Encryption (ABE) security technique can provide an authentication protocol for encrypted data by applying private keys to form cypher-text and by using access control policies on the required attributes^2^. The authors in^3^ proposed an ABE that provides encryption as well as a fine-grained authentication mechanism. Key Policy with Attribute Based Encryption (KP-ABE) and Ciphertext creation Policy through Attribute Encryption (CP-ABE) are two types of attribute-based encryption methods that were used in^3^ to encrypt the data. The KP-ciphertext ABE and decryption key are bound to the access policy, while the CP-ABE ciphertext and decryption key are linked to attributes. In order to enhance the security through high level attribute operations and also by applying more flexible access control policies, many ABE schemes have been proposed in the past in the provision of secured communication which includes the use of access control list with ABE. Moreover, the type of possible attacks and the methods for prevention are also discussed with respect to the handling of sensitive information^4^. All of these methods that are available in the literature on security are also prone to security attacks due to the presence of malicious users.

In order to handle the problem of key leakage that is used in encryption and decryption, we propose a secured key exchange scheme through the application of a new bilinear group based Diffie–Hellman approach for key generation which is used with RSA algorithm for effective encryption of the data to be communicated. Moreover, we also propose a new trust-enabled secured routing scheme which is used to secure the WSN communication from the attacks of malicious users. In this model, we form clusters and select a Cluster Head (CH) for each cluster and we choose a set of trusted CHs for routing more securely. The major contribution of this model includes security enhancement through enhanced cryptographic techniques and trust measurements, energy efficiency through clustering and improvement in network performance based on secure routing. Through wireless sensor nodes provide protection against malicious attacks on the network by applying firewalls and authentication methods, the security challenges are still existing in WSN. In this way, we propose a new routing algorithm that applies clustering and trust measurement and decision-making model to form the secure routing algorithms in combination with data encryption. Here, improved route discovery and route maintenance techniques are proposed by considering the trust values so that it is possible to make the proposed clustered-based routing algorithm more efficient in WSNs. The proposed secure routing protocol has a unique ability to prevent malicious attacks because of its operations and maintenance policies which are reliance on trust evaluation. In the proposed cluster and trust base secure routing algorithm, routing is performed through cluster heads. Here, the route discovery process evaluates the trust values for selecting the next node for forwarding the data.

This trust-based secure routing model is using the broadcasting feature present in the proactive based routing protocols that is providing the secured and trusted neighbour node to assign as the next hop as it is followed in^5^. In the proposed security model, the system ensures trust between the sender and the receiver through message exchanges before they start the actual communication. In this way, the network is able to identify the genuine as well as malicious nodes and use that information to provide the secured routing process by selecting the paths through the trusted and active nodes.

In this work, we propose a new ABE-TBSRA scheme based on bilinear pairing and Diffie–Hellman encryption scheme with RSA algorithm in order to maintain the confidentiality of the data. Along with this method, we propose a trust management scheme for developing the secure routing process which is used to send the data from source to the Base Station (BS) more securely. Moreover, clustering technique is used for formation of clusters along with cluster head selection in WSN. In this research work, we compute the trust score of nodes and paths which are computed based on the encryption and decryption attempts by the nodes in order to improve the reliability of routing process in wireless sensor networks by adding better security and functionality. This proposed method is particularly successful at finding solutions to the security issues by compromising the integrity which are introduced by the attackers that appear when transmitting data. This proposed model uses attribute-based encryption based on bilinear pairing and Diffie–Hellman scheme with RSA algorithm and we introduce a new key management technique for finding more reliable and secure routes in wireless sensor networks. Furthermore, this work examines the use of direct, indirect, and overall values of the computed trust obtained through the proposed model for enhancing the security of WSN communication.

The main and important contributions of this proposed work are as follows:

- A new ABE-TBSRA has been proposed and implemented in this research work in order to reduce and optimize the energy consumption of the nodes and also the delay occurred in WSN communication.
- The proposed Attribute based encryption method uses the properties of bilinear pairing groups to secure the data communication by providing new key generation methods.
- For exchanging the keys between the sender and receiver, Diffie–Hellman encryption method with bilinear pairing scheme and RSA algorithm is used in the proposed algorithm ABE-TBSRA.
- The use of clustering of nodes in WSN and also performing the routing through CHs improves the network’s resilience through reduction in hop count and also with respect to energy consumption in the routing process.
- A new trust management scheme has been proposed in this paper for providing improved security in the routing process for WSN communication.

## Related works

There are numerous works which are present in the literature on security and secure routing algorithms^6,7^. Since security is a major concern in WSNs, the proper handling of security is necessary either using encryption and decryption methods or using trust management techniques. The currently available data encryption and decryption methods have either extremely high response times or extremely high computational costs, making them unsuitable for use in the securing of wireless sensor networks. In the past, various techniques were proposed to secure the communicated data with reliability to overcome the problems related to energy, coverage, security, and routing. A wide range of techniques are discussed in the literature for providing secure routing of data in WSN.

Han et al.^8^ propose an energy-aware and trust-based secure routing protocol for wireless sensor networks (WSNs) leveraging an adaptive genetic algorithm. The protocol optimizes routing by balancing energy efficiency and trustworthiness among nodes, ensuring secure and reliable data transmission. The adaptive genetic algorithm dynamically adjusts to network conditions, enhancing performance while extending the network’s lifetime. This approach addresses key WSN challenges, including energy constraints, security risks, and maintaining consistent communication quality.

Aravindan and Rajaram^9^ present an energy-aware multi-attribute trust model designed for secure communication in MANET-IoT environments. The model evaluates nodes based on multiple attributes, such as energy efficiency and trustworthiness, to ensure reliable data transmission and robust security. By integrating energy-awareness into trust assessment, the approach extends network lifetime while mitigating risks from malicious nodes. This solution is tailored to address the unique challenges of MANET-IoT environments, including dynamic topology, resource constraints, and security threats.

Zhang et al.^10^ propose a secure routing strategy based on attribute-based trust access control for social-aware networks. The approach leverages attribute-based trust models to evaluate and select routing paths, ensuring that only trustworthy nodes are involved in data transmission. By incorporating social-awareness, the strategy accounts for the relationships and behaviors of nodes in the network, enhancing security and reliability. This method aims to mitigate security risks, such as attacks and data breaches, while optimizing routing efficiency and trust management in dynamic network environments.

Feroz Khan^11^ presents an Enhanced Multi-Attribute Based Trusted Attack Resistance (EMBTR) model for secure routing of sensor nodes in WSNs. The model incorporates multiple attributes, such as energy levels, trustworthiness, and node behavior, to assess and select the most reliable routing paths. EMBTR enhances network security by identifying and resisting attacks while ensuring efficient communication. The approach is designed to handle the unique challenges of WSNs, such as resource constraints and vulnerability to malicious nodes, offering a robust solution for secure data transmission in these networks.

Khan and Rajalakshmi^12^ propose a multi-attribute-based trusted routing approach for embedded devices in MANET-IoT environments. The model evaluates various attributes, such as energy, trust, and connectivity, to ensure the selection of reliable and efficient routing paths. By integrating trust management with multi-attribute analysis, the approach enhances the security and performance of communication in dynamic, resource-constrained networks. This method addresses key challenges in MANET-IoT systems, including node mobility, limited resources, and the need for robust data transmission, providing a secure and efficient solution for embedded devices.

Yesodha et al.^13^ introduce an elliptic curve encryption-based energy-efficient secured Ant Colony Optimization (ACO) routing protocol for WSNs. The protocol combines the advantages of elliptic curve encryption for data security with the energy-efficient capabilities of ACO to optimize routing paths. It ensures secure data transmission while minimizing energy consumption, addressing the challenges of energy constraints and security in WSNs. This approach enhances network lifetime, improves security, and ensures reliable communication in dynamic and resource-limited environments like WSNs.

Rani et al.^14^ provide a comprehensive survey on attribute-based encryption schemes for next-generation wireless IoT networks. The paper explores various encryption techniques that enable fine-grained access control, where data access is determined by specific attributes or characteristics of the user. These schemes offer enhanced security for IoT networks by ensuring that only authorized users with the correct attributes can access sensitive information. The survey also examines the challenges and potential solutions in implementing attribute-based encryption in dynamic and large-scale IoT environments, focusing on scalability, efficiency, and privacy.

Dinesh and Santhosh Kumar^15^ propose an energy-efficient, trust-aware, and secure neuro-fuzzy clustering technique for wireless sensor networks (WSNs) using sparrow search optimization. The approach combines the benefits of neuro-fuzzy systems for clustering with sparrow search optimization to enhance energy efficiency and security. It incorporates trust management to ensure secure data transmission while minimizing energy consumption. The proposed method aims to optimize network performance by balancing clustering accuracy, energy efficiency, and trustworthiness, making it well-suited for resource-constrained WSNs.

Diarra and Islam^16^ introduce an energy- and trust-aware routing approach for wireless networks to optimize multimedia data transmission. By selecting nodes with higher energy and trust levels, the model reduces energy consumption, enhances security, and ensures reliable data delivery. This approach improves the quality of service (QoS) for multimedia applications by addressing challenges like bandwidth demands, data integrity, and latency.

In^17^, the authors proposed an attribute-based encryption technique, and they send the encrypted data using this advance cryptosystem, since it supports the key establishment values, and it also increases the productivity of key concerns in the formation of ciphertexts. Jie Ling et al.^18^ proposed a multi-authority and attribute-based encryption scheme that is having traceable as well as dynamic updating policies by using finite groups to handle the domain attributes, expressive power and finally security in WSN.

In^19^, the authors proposed a new method for securing the data communicated through the network and stored in a repository that can be used for private data retrieval based on the cypher text policy of attribute-based encryption techniques proposed by them. In their model, the trust values are evaluated across all the nodes of the network, and it is updated in the trust table on a regular basis. From the experiments carried out by them, the authors have proved that their model significantly improved the performance of the network by reducing the communication cost. According to the authors, the use of trust mechanisms and encryption policies with intelligent agents was a limitation of their research work. Shi et al.^20^ proposed a new ABE encryption scheme for handling the security issues by encrypting the plain text by applying their scheme since it provides a structured method in storage of data and hence must be encrypted with similar structure. They applied their algorithm and compared it with Chinese remainder theorem-based encryption method. In^21^, the authors have provided a detailed review on the use of attribute-based encryption in cloud databases for secure storage. They proved that their method provided security in searching and storing the information in the cloud database more efficiently. In^22^, the authors proposed a new key distribution model using Elliptic Curve and Diffie–Hellman based key exchange mechanism that allows the sending user to satisfy the required security mechanisms. In their model, they improved the security and reduced the energy consumption. Kwon et al.^23^ have proposed a novel Device to Device (D2D) authentication mechanism which used the Ciphertext-Policy (CP) based encryption/decryption method for protecting the key generation process. The major advantages of their security model include the increase in reliability and security through cryptographic approach.

In^24^, the authors proposed a new method for protecting the privacy of the CP-ABE scheme using the keyword-based searching techniques. According to the authors, their model is more suitable for use with limited resources and they provided the security using bilinear Diffie–Hellman expectation algorithm. Sabrina Sicari et al.^25^ provided a critical analysis by comparing the existing CP-ABE with other sticky policy methods for improving the security of data in their application. In^26^, the authors proposed a new encryption method based on ABE and they claimed that their method is resistant to traffic snooping, sensor concessions, and the use of unauthorised data access. They used a Merkle tree-based data structure for modelling the network. Mriganka and Mandal^27^ proposed a ciphertext policy-attribute-based broadcast authentication scheme which provides full anonymity through the modification of an identity-based encryption structure over bilinear pairings for enhancing security in communication.

In^28^, the authors have developed a new trust aware and low-energy based adaptive clustering protocol. Their protocol was able to enhance the energy performance and also it avoided the elevated node power consumption problem. The major uses include enhancement in security and network lifetime. In^29^, the authors proposed a realistic and trust-aware, secured routing algorithm to detect and prevent the malicious nodes from participating in the communication process using a hybrid trust model. They performed a statistical analysis and carried out packet switching through multivariate regression analysis. In spite of the presence of many encryption schemes and trust mechanisms, the security strength of the existing algorithms is not able to maintain the necessary levels in sensitive WSN applications.

In^30^, authors have proposed an efficient attribute based revocable encryption scheme which ensures data integrity during message transmission. Their scheme employs ABE which encrypts the data by using attributes to maintain integrity during message exchange and it can be revocable. The advantages of the scheme are it performs efficient encryption of messages and prevent the intruders to intercept the messages during the transmit. However, the limitations of the scheme are it suffers from overhead in terms of communication and computation.

In^31^, authors have proposed an efficient policy based multi-party attribute based encryption scheme to ensure efficient and secured cloud storage. In their scheme, they have formulated the policy for multiparty attributes to ensure efficient encryption of data during the storage and also in transmit in the cloud environment. The advantages of the scheme are providing an efficient security mechanism in the cloud environment. The limitations are key revocation is not addressed in their scheme.

In^32^, authors have proposed an efficient Cipher Text Policy based ABE scheme with shared decryption in cloud storage. In their scheme, the messages are encrypted and decrypted by using CP based ABE scheme. The main advantages of the scheme are efficient encryption and decryption of the messages with less computational time. However, the limitations are it is prone to various types of security attacks which are caused by the various intruders. In^33–35^, authors have proposed an efficient multiparty based fine grain ABE scheme for ensuring efficient security during message transmit in the network. The main advantages of the scheme are providing efficient encryption and decryption process with better communication and computation overhead. However, their schemes are most likely to be prone from various types of security attacks.

Based on the analysis of the literature survey, most of the existing schemes does not provide trade-off between energy efficiency and security. In most of the schemes available in the literature, they only either focuses on energy optimization for the nodes of WSN or providing security to the nodes of WSN. The major research gap identified in the literature survey is most of security schemes suffers from high computation overhead and they are vulnerable to various types of attacks caused by the intruder. Moreover, due to the resource constraint nature of WSN, there is a high requirement of light weight trust based authentication schemes which are required to provide better security with optimized energy consumption. Therefore, we propose a more efficient ABE encryption scheme by extending the ABE schemes along with trust-based routing for enhancing the communication security.

## Proposed methodology

**Fig. 1:**
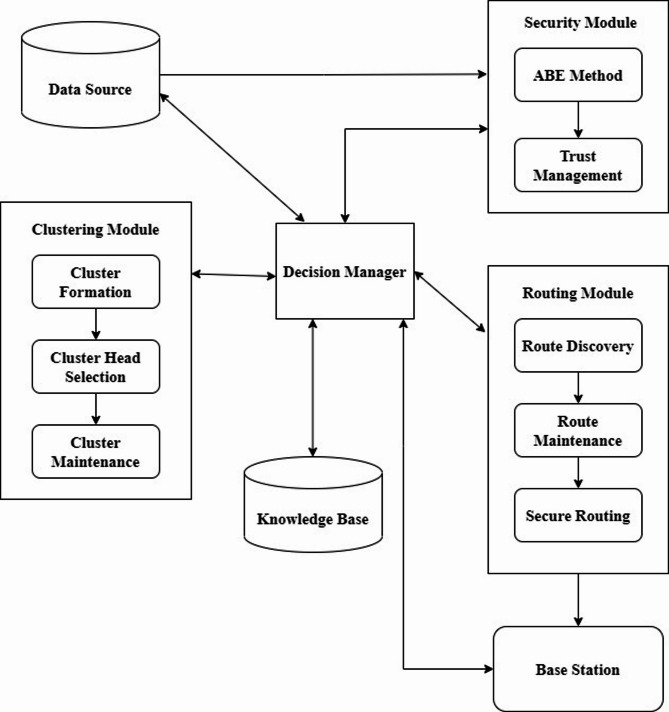
System architecture.

In this paper, we have proposed an effective Attribute based Encryption scheme along with a Trust model for measuring the characteristics of nodes to develop the proposed secure route algorithm called ABE-TBSRA. This algorithm uses ABE for encryption, trust measurement for selecting secure nodes and finally we use bilinear pairing with Diffie–Hellman algorithm for key generation. Moreover, the keys are used in the RSA algorithm for enhancing of the communication security in WSN. Figure 1 gives the components of the proposed system which consists of the major modules namely data source, security module, clustering module, routing module, decision manager and knowledge base.

The functions of the system have been detailed in this section. Here, the data which are collected by the sensor nodes are first encrypted by applying the ABE approach. Moreover, clustering and CH selection are carried out for improving the energy efficiency. All the modules shown in this architecture are monitored and controlled by the decision manager. We use the security module to perform key generation and exchange, encryption/decryption and also trust measurement and management. There is a knowledge base in the system that provides the security rules and attacker related rules for monitoring the communication through inference. The routing module is coordinating with the other modules by the direction of the decision manager for secured delivery of data collected from the sensor nodes to the sink node as it is done in^36,37^ with suitable security enhancements.

### Key generation

A generation of keys takes the authority of the system by using master keys with the set attributes are used as the input by the user and the secret key as the output in the encryption system. It is initially executed by the decision manager present in the base station of the wireless sensor networks by the use of a sub component namely the Key Generator Admin (KGA). Here, the KGA issues a set of secret keys initially that correspond to the attributes of each node. It assumes that the proposed model first sets-up the network initially and encrypts the data to be communicated through the network using key generation method^38^. In addition, the KGA makes public key parameter as pk and a master key parameter as mk. The KGA attains the necessary permissions from the database manager and then it confirms to the distribution of the keys more effectively with the cluster head nodes. Next, it generates a key to be used between two parties using the Diffie–Hellman encryption scheme with RSA algorithm and it is communicated through the network. In the key generation phase, two parties namely Alice and Bob who need to construct the transmission values of g and h which are to be prime numbers. The communication values are calculated by using this formula as p = H mod G, where p is the remainder obtained from H mod G operation. Now, the two parties exchange control packets for purpose of knowing each other. In this work, new values are generated by using the formula and the keys are known by both parties are G and H, then the private key is generated by the decision manger and the secret keys are passed to both the parties with the help of security manager. Now, A chooses a value x and computes the value of p^x^ and B chooses the value of y and computes the value of p^y^. now, A sends p^x^ to B and B sends p^y^ to A. A and B compute the values of c = (p^y^)^x^ and d = (p^x^)^y^. Since both are producing the same value, they are verifying the values of c and d for validating their keys. These keys are used along with RSA algorithm for effective encryption of the first attribute. This procedure is repeated for all the attributes by considering a set of x values namely x_1_,x_2_,..,x_n_ and set of y values namely y_1_, y_2_,…,y_n_ for encrypting n given attributes. The verification process is based on the values of c with c_1_, c_2_,..,c_n_ and d with d_1_,d_2_,.. d_n_. If the values are matching the RSA based encryption is performed by the respective keys of A and B for encrypting their attributes before performing the communication.

### Key distribution, encryption and decryption

The key distribution technique provides a user A with an encryption key ek(x) and the receiving user B with a decryption key called dk(x). If the new user joins the group and then the old user decides to get a new decryption key because the existing key has been expired or it has been negotiated. In this model, a trusted third party is present in the base station of the network for performing key generation and key distribution more effectively. Moreover, the trusted third party first defines the access policy for the sender A by generating new user key called x. The trusted third party of the proposed system is responsible for applying the access policy of the application for generating the required keys for encryption and decryption and then by assigning them to each user. Once the trusted third party has well-defined the users access control policy p^x^, it creates the sender’s encryption key ek(x) and forms the decryption key dk(y) by executing the following equation.

1 $$ek(x) = KG(p^{x} ,p^{y} ,\phi (xy),d,e)$$

2 $$dk(y) = KG(p^{x} ,p^{y} ,d,e)$$

where KG is the key generation function used for all the nodes in the cluster formed in this work from the nodes of the WSN. The trusted third party encrypts the message using the equation.

3 $$CT = Enc(PT,ek(x))$$

Here CT is the cipher text, $\phi (x)$ is the Euler’s phi function and PT is the plain text. Moreover, the encryption process calls the RSA algorithm with Euler’s phi function. The message is sent from the sender side to the receiver’s side namely the base station in encrypted form. The received message is decrypted at the base station using the equation.

4 $$PT = Dec(CT,dk(y))$$

Here, the verification of the users is performed using Diffie–Hellman key exchange method with RSA algorithm along with access policy of the application with the support of the trusted third-party present in the base station.

### Trust computation

The trust values are combined into the cluster heads for selection process. The trust value of a nodes in a cluster is taken into account by the CHs nodes in the present round at secure routing process. Nodes with a high degree of trust are more likely to be chosen as path discovery transmission relays^39^. Trust values consist of three parts namely direct, indirect and overall trust values.

#### Direct trust value

In this work, a direct trust model computes every cluster in its own value called Direct Trust (DT) computation is based on the composed and direct packages to its neighbours^40^, which allows it to calculate its implication with each neighbour and current DT of node a, then the actual degree of trust with respect to its neighbor b node, which is given by equation.

5 $$DT_{ab}^{n} = \alpha * (\beta_{1} rcvd_{p} + \beta_{2} snd_{p} )^{n - 1} + (1 - \alpha ) * (rcvd_{p} + snd_{p} )^{n}$$

Here, $DT_{ab}^{n}$ stands for direct trust value of node n obtained by sending the message from the user a to user b. the values of this value are more important for effective decision making. In the former scenario historical trust value, it denotes the trustworthiness in the past^41^, whereas the later current trust value means that reliability now.

6 $$rcvd_{p} = \frac{{receive\_message_{b} }}{{message_{b} }}$$

$\alpha$ and $(1 - \alpha )(0 < \alpha < 1)$ are the weights that correspond to history and current trust values and it can vary with individual uses of WSNs. Finally, $rcvd_{p}$ and $snd_{p}$ are the ratio of sent and received packets over total packets^42^ which is represented as

7 $$snd_{p} = \frac{{send\_message_{b} }}{{message_{b} }}$$

Additionally, volatility or uniqueness factor $\beta_{1}$ and $\beta_{2}$ have been established to expeditiously filter out malevolent nodes, which were once normal sensor nodes were increase their trust levels. In order to return a previous trust level to a normal range^43^, the formulas shown in equation.

8 $$\beta_{1} = e^{{ - f_{1} \bmod (T,\lambda )}}$$

9 $$\beta_{2} = e^{{ - f_{2} \bmod (T,\lambda )}}$$

where T is the present time of the system and $\lambda$ is the time threshold. Next, the $f_{1}$ and $f_{2}$ are factors used to regulate the rate of change in trust value. In order to avoid miscategorized valid nodes, like those that have been off of the network in distribution and forwarding packets for a long time are mod (T, $\lambda$), the training part is strengthened with trust management. To keep track of the past trust values which are becoming too tiny or to make sure that volatile factors degrade over time in a particular range, the proposed algorithm uses temporal rules. Moreover, the values of $\lambda$, $f_{1}$ and $f_{2}$ are application-specific.

#### Indirect trust value

The third-party network node trust relationship is utilised in the computation of Indirect Trust (IT) values for nodes a and b. The node $s_{a}$ has a reputable status because it belongs to a third party. Node a is a trusted assessor, node b is for calculating the target and node $s_{a}$ is the recommended of sensor node a. Here, $s_{a} \in s_{p} = \{ s_{1} ,s_{2} , \ldots ,s_{c} \}$, where c is the common trusted nodes and $s_{p}$ is the set of public trusted neighbor nodes of node a and b. The IT value of node a to node b is shown in^44^ based on the relationship given in equation.

10 $$IT_{ab}^{n} = \frac{1}{c}\sum\limits_{{s_{a} \in s_{p} }} {(DT_{{a * s_{a} }}^{n} } * DT_{{s_{a} * b}}^{n} )$$

where $DT_{{a * s_{a} }}^{n}$ is the direct trust value of node x to $s_{a}$ and $DT_{{s_{a} * b}}^{n}$ is the DT of node $s_{a}$ to b, where node $s_{a}$ is any public trusted neighbor of a and b. Node $s_{a}$ will be removed from the set of public trusted neighbors if the trust value of node a to $s_{a}$ is less than threshold.

#### Overall trust value

The Overall Trust (OT) values are computed the overall performance of direct and indirect values with their weight factors. The nodes are communicated with a third-party trust management scheme. Finally, the nodes are transmitted the data through the trust factors with more reliable and secure in their routing process^45^. Finally, the overall trust value is computed using the formula:

11 $$OT = ((w1 * DT) + (w2 * (\beta_{1} + \beta_{2} )) + (w3 * IT) + (w4 * \alpha ))/4$$

In this work, the value of OT must be greater than or equal to 0.65 for a node to be recognized as a genuine node for communication.

### Clustering and cluster head selection

The routing performance in wireless sensor networks can be extended by using clustering techniques. In this work, we propose a cluster-based routing algorithm and hence it has a mechanism for selecting the CH in each round. Since, it takes more energy to re-cluster, clustering often will reduce the network’s life expectancy^46,47^. Therefore, this work performs CH rotation only after the energy level and trust level of the CH goes below a threshold value. In this model, clustering is accomplished by dividing the network into a number of unequal groups and hence the clustering process is validated using rules. Using the proposed clustering and cluster-based secure routing algorithm, the CHs are selected for each of the clusters from the cluster members node with minimum distance, high energy, low mobility and also with trust values^48^. When two or more nodes have the same transmission power, the nearest node to the cluster’s centre is selected as the new CH. The CH selection and rotation are carried out in this work using a mathematical model based on the computation of a threshold value, THV(a). Cluster members of a cluster will decide on whether a new CH is needed for the current round based on the current threshold value during every round of the cluster-based routing process^49^. If a new node has the possibility of becoming a cluster member in a cluster working under a CH, it can choose its own cluster from the nearest clusters based on distance. In order to support in decision-making, each new node is assigned a trust value between 0 and 1. If the value is greater than the variable THV(a) in the equation, then the current node will become a possible candidate node for inclusion in the next round. The value of THV(a) for a node a is computed using the formula

12 $$THV(a) = \left\{ {\frac{\text{P}}{{1 - P * \left( {r\bmod \frac{1}{P}} \right)}}} \right\}if;_{4} a \in s$$

where s represents the set of sensor nodes that did not change from CH to non-CH in the preceding round with the value 1/P. Here in round r, the percentage of CHs in a network is considered as P, n is the total number of nodes, and r is the present round. The function, Mod () returns the remainder after a number is divided by the divisor, which ensures the nodes with equal opportunity to become the CH.

### Cluster and trust based secure routing algorithm with energy efficiency

In this work, we proposed a novel ABE-TBSRA attribute-based encryption strategy by combining bilinear pairing and Diffie Hellman and RSA based encryption scheme to encrypt and secure the data. This algorithm provides a method for calculating the key management for encrypting and decrypting the data with most secure, and quickest way to transfer the data packets from the source to the destination. This protocol is an optimized routing protocol in which the nodes are clustered and it provides an optimal path for effective routing in WSNs. In this section, we have given a complete description about our proposed ABE-TBSRA method. In this model, bilinear mapping is used to construct the keys using Diffie–Hellman key exchange with RSA algorithm. Moreover, we consider two cyclic groups namely G and G_m_ to form the bilinear pairing. The main advantage of using bilinear pairing along with Diffie–Hellman algorithm with RSA algorithm is to enhance the security by making use of the difficulty present in the discrete logarithm problem.

In this model, the trusted third-party present in the base station is responsible for selecting the groups G and G_m_ along with the generator g for the cyclic groups. This value of g is used to compute the values of g^x^ by the sender and g^y^ by the receiver. Key sharing is accomplished by the combination of bilinear pairing with Diffie–Hellman algorithm. In this proposed ABE scheme, separate keys are computed for each of the attributes namely a_1_, a_2_,..,a_n_. For each attribute the sender is selecting a prime number x and hence the sender selects a set of prime numbers namely x_1_, x_2_,..x_n_ and the receiver also selects a set of prime numbers y_1_,y_2_,..,y_n_. These values are used for generating the keys for encryption and decryption using RSA algorithm and they are also used for key verification and user authentication by applying bilinear Diffie–Hellman scheme with RSA algorithm. The algorithm for our proposed ABE-TBSRA method are as follows.

**Algorithm 1:**
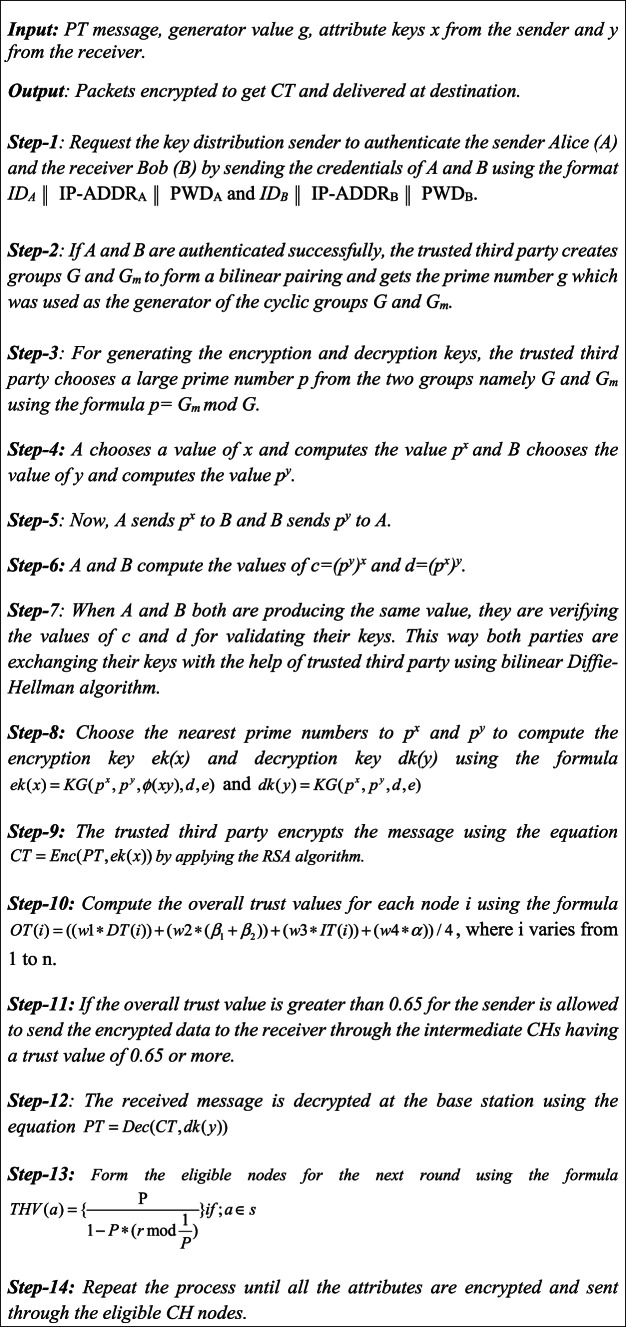
ABE-TBSRA Algorithm

## Security analysis

Formal security analysis plays a crucial role in ensuring secure data communication within WSN by rigorously evaluating the security properties and protocols involved. This involves using mathematical frameworks to validate confidentiality, integrity, authentication, and availability of data transmitted across the network. Security properties such as non-repudiation are formalized to ensure that entities involved in data communication cannot deny their actions. Moreover, access control mechanisms are verified using formal models to ensure that only authorized nodes can access sensitive data during routing and aggregation processes.

The analysis also assesses the robustness of data communication protocols against threats like insider attacks, Sybil attacks, and replay attacks. By leveraging formal methods, researchers can establish a mathematically sound foundation for security claims, offering higher assurance against vulnerabilities. Such analysis forms the cornerstone for designing secure and efficient routing protocols in WSNs, especially in applications requiring stringent data confidentiality and integrity.

In the proposed secure data communication approach, efficient encryption techniques are employed to safeguard routing in WSNs. The columnar transposition cipher method is used to establish secure communication between sensor nodes and their cluster head nodes, while the Residue Number System (RNS+) technique enhances security in communications between CH nodes. The security analysis focuses on two key aspects: correctness and confidentiality. Correctness ensures that the sink node accurately receives the data after applying encryption techniques, while confidentiality prevents unauthorized access to sensitive information during routing.

### Theorem 1

The proposed secure communication method ensures that the sink node receives data accurately after encryption.

### Proof

The proof is based on an attack model where attackers are limited to eavesdropping on communication channels without the ability to modify or replace messages. The encryption algorithm ensures that CH nodes encrypt data using a cryptographic key before transmission. The CH node decrypts these encrypted messages to retrieve data from its associated nodes. Since the cluster key is exclusively shared among legitimate nodes within the cluster, it remains inaccessible to unauthorized entities. This secure key-sharing mechanism guarantees the correctness of data communication within the cluster.

### Theorem 2

The proposed method ensures the confidentiality of data during communication.

### Proof

During data communication and aggregation within a cluster, encryption renders the content inaccessible to attackers without the cryptographic key. Additionally, obtaining unique parameters such as sensor node IDs, hash messages, and encrypted cryptographic keys is infeasible for attackers. These parameters are exclusively known to the base station and the respective CL nodes, adding another layer of security. The use of diverse, node-specific parameters ensures robust confidentiality.

Furthermore, the proposed method is resilient to eavesdropping, traffic analysis, and node capture attacks. Encrypted data and secure key management mechanisms make it challenging for attackers to extract meaningful information, ensuring secure and efficient routing in WSNs.

### Informal security analysis

The informal security analysis demonstrates that the proposed system effectively counters a variety of threats, including Sybil, replay, eavesdropping, and node capture attacks, while maintaining data confidentiality, integrity, and authenticity. By combining efficient encryption techniques, secure communication protocols, and proactive defence mechanisms, the system ensures reliable and secure data communication in WSN routing. An informal security analysis for the proposed system would focus on evaluating the system’s ability to resist common security threats in a practical and conceptual manner, without relying on formal proofs or mathematical models.

#### Resilience against Sybil attacks

The system employs robust authentication and validation mechanisms to verify the legitimacy of each node participating in the network. By using secure key management, digital signatures, and reputation systems, it ensures that malicious nodes cannot create multiple fake identities to disrupt the network. Additionally, anomaly detection algorithms actively monitor the data for irregular patterns, effectively identifying and mitigating Sybil attacks. These features bolster the system’s ability to maintain trustworthiness and integrity in routing operations.

#### Prevention of replay attacks

The system incorporates timestamping and nonce values to verify the freshness of each message. This ensures that repeated or outdated data is automatically discarded. Furthermore, the use of secure authentication mechanisms guarantees that only legitimate nodes can send or receive data. Since the system authenticates every message entering the network, it effectively blocks replay attempts and maintains the integrity of the routing process. This makes the system particularly resilient to time-based attacks where attackers attempt to reuse valid data packets.

#### Protection against eavesdropping attacks

To guard against unauthorized interception, the system employs strong RSA encryption technique. These encryption methods ensure that even if communication is intercepted, the data remains unintelligible to attackers. Additionally, the secure communication channels used between nodes and clusters further enhance data confidentiality. This proactive approach ensures that sensitive information, such as routing paths or sensor data, remains protected from prying eyes, preserving privacy.

#### Defence against node capture attacks

Node capture attacks pose a significant threat in WSNs, as attackers can compromise nodes to extract sensitive keys or disrupt network communication. The system mitigates this threat by limiting the exposure of cryptographic keys. Only legitimate cluster nodes and the base station have access to the necessary encryption parameters, ensuring that even if a node is compromised, the overall network security remains intact. The system’s hierarchical design minimizes the impact of such attacks by isolating compromised nodes and maintaining secure communication among unaffected nodes.

#### Secure routing and data integrity

The system ensures secure routing by implementing encryption at multiple levels. Sensor nodes encrypt data before sending it to the cluster head, and further encryption is applied during inter-cluster communication. The hierarchical use of encryption prevents unauthorized modifications to the data while ensuring that the base station receives accurate and reliable information. This approach also guards against data tampering during transmission, preserving the integrity of routing and aggregation processes.

#### Mitigation of insider threats

Insider threats, where malicious nodes within the network attempt to disrupt operations, are addressed through efficient authentication and authorization mechanisms. Only nodes with valid credentials can participate in the network, and the distributed nature of the system ensures that malicious nodes are quickly identified and isolated. The system’s anomaly detection further reinforces its defences by flagging suspicious activity from internal nodes.

#### Handling traffic analysis

To mitigate traffic analysis attacks, the system encrypts not only the data but also key routing information. This obfuscation prevents attackers from analysing communication patterns to infer sensitive details, such as node locations or the frequency of data transmission. By employing secure communication protocols, the system minimizes the risk of information leakage through traffic analysis.

## Results and discussions

In this paper, a proposed ABE-TBSRA method has been simulated by using NS-2 simulator. Table 1 shows the parameters that are used for simulation of the secure routing experiments in WSNs carried out in this work. The experiments have been conducted in this work, the proposed method is evaluated and its performance is compared with existing algorithms are CKM-ABE^8^, LEACH-TM^19^ and TASRP^20^.

**Table 1:** Simulation parameters.

Parameters	Value
Network area	200 m × 200 m
Number of sensor nodes	100–1000
Basic routing protocol	LEACH
Initial energy oof nodes	2Joules
Mobility model	Random way point mobility

### Security level with encryption

**Fig. 2:**
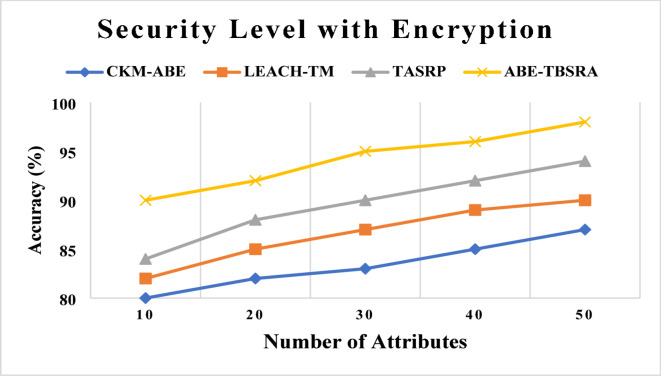
Security level with encryption.

The proposed ABE-TBSRA algorithm has been implemented and has been evaluated by comparing with three existing protocols namely CKM-ABE^8^, LEACH-TM^19^ and TASRP^20^. The evaluation has been carried out with several numbers of attributes such as 10, 20, 30, 40 and 50. The security level comparison of the proposed algorithm and the existing algorithms are shown in Fig. 2.

**Fig. 3:**
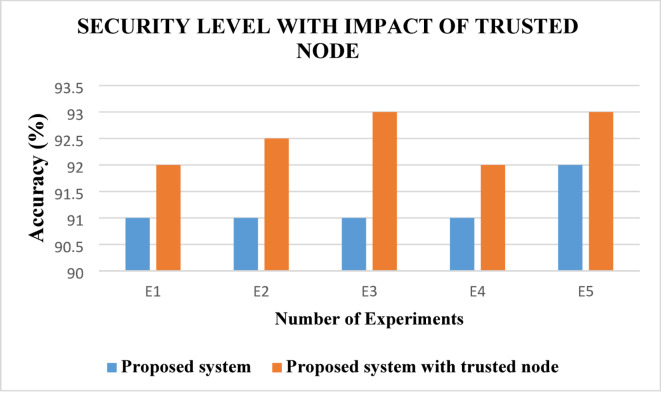
Security Level with impact of trusted nodes.

From this Fig. 2, it observed that the proposed algorithm namely ABE-TBSRA is higher in their security level with encryption techniques, when it is compared with other existing protocols namely CKM-ABE^8^, LEACH-TM^19^ and TASRP^20^. The improvement in security level is obtained in this work by choosing keys with bilinear pairing, taking large prime numbers based on *p*^*x*^ and *p*^*y*^ values, use of trusted third party for key generation, key exchange using bilinear paired Diffie–Hellman algorithm and RSA based encryption before routing. Moreover, trust management is also performed in this work for improving the secure routing further. Figure 3 presents the proposed system’s security level in comparison with the trusted nodes.

### Packet delivery ratio

**Fig. 4:**
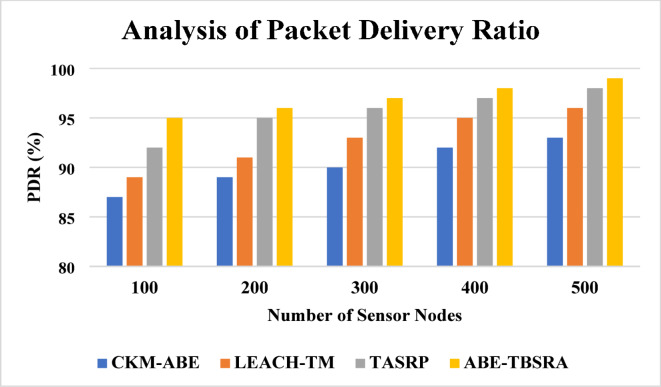
Analysis of packet delivery ratio.

**Fig. 5:**
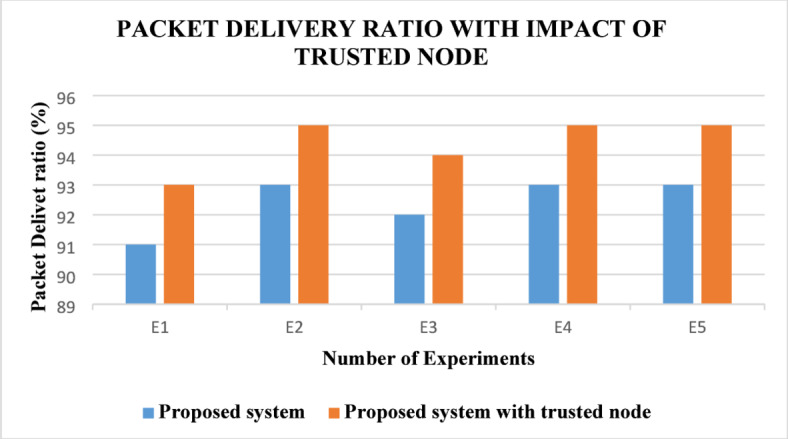
Packet delivery ratio with impact of trusted nodes.

Packet delivery ratio analysis are shown in Fig. 4. Here, the proposed ABE-TBSRA algorithm has been related with the three existing protocols namely CKM-ABE^8^, LEACH-TM^19^ and TASRP^20^. The experiments have been carried out with several numbers of sensor nodes such as 100, 200, 300, 400 and 500. Figure 5 presents the proposed system’s packet delivery ratio in comparison with the trusted nodes.

From Fig. 4, it is perceived that the packet delivery ratio of the proposed ABE-TBSRA is higher than the existing protocols namely CKM-ABE^8^, LEACH-TM^19^ and TASRP^20^. In this work, the packet delivery ratio is increased by performing suitable clustering and re-clustering periodically and also by rotating the cluster heads. Moreover, the trust management method proposed in this work enabled the detection of packet dropping attacks and hence the packet delivery ratio has been improved.

### Analysis of delay

**Fig. 6:**
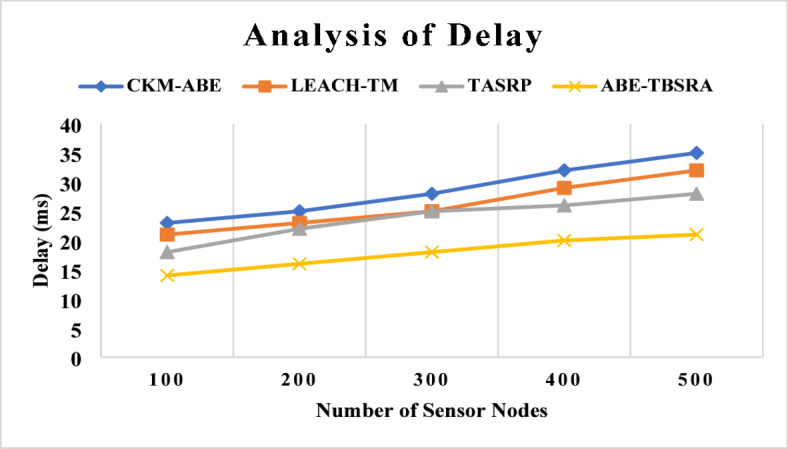
Analysis of delay.

The proposed ABE-TBSRA algorithm has been evaluated by conducting the experiments on the delay analysis by considering the values of delay between the ABE-TBSRA method and compared with other protocols namely CKM-ABE^8^, LEACH-TM^19^ and TASRP^20^. The experiments have been carried out with a greater number of sensor nodes such as 100, 200, 300, 400 and 500. The analysis of delay is shown in Fig. 6.

**Fig. 7:**
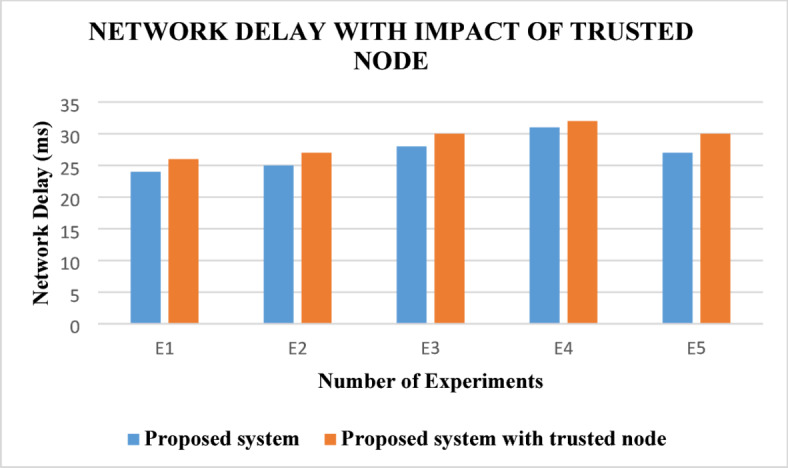
Network delay with the impact of trusted nodes.

From this Fig. 6, it can be observed that the ABE-TBSRA algorithm has decreased the delay when it is compared with the other methods namely CKM-ABE^8^, LEACH-TM^19^ and TASRP^20^. In addition, the proposed algorithm has been made secured routing using direct, indirect and overall trust values. The reliability of the communication was ensured through security, leading to better route selection and fast communication, leading to reduction in delay. Figure 7 presents the proposed system’s network delay in comparison with the trusted nodes.

### Security with encryption and trust modelling

**Fig. 8:**
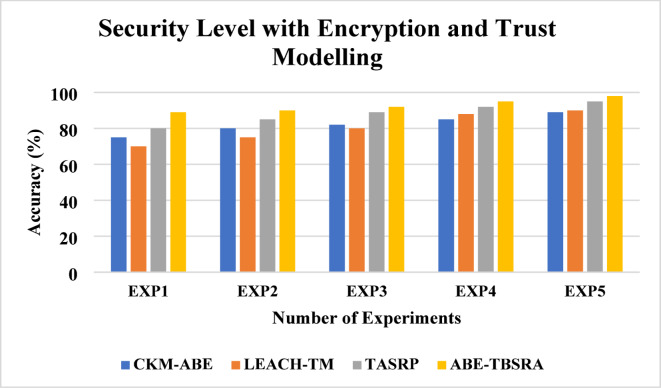
Security level with encryption and trust modelling.

The analysis of overall security with encryption and trust modelling is shown in Fig. 8. Here, the proposed ABE-TBSRA algorithm has been evaluated by conducting the experiments on the security with encryption of data packets before transmitting the packets and then improving the security further using trust-based secure routing method. Moreover, the proposed algorithm namely, ABE-TBSRA has been compared with other three existing protocols namely CKM-ABE^8^, LEACH-TM^19^ and TASRP^20^.

From Fig. 8, it observed that security level with encryption and trust modelling provides increased security when it is compared with the other methods namely CKM-ABE^8^, LEACH-TM^19^ and TASRP^20^. The improvement is higher than only trust modelling because the copying of data packets is not useful to the malicious users since the data packets are sent in encrypted form. This leads to increase in copying type of passive attacks in addition to the reduction in active attacks through trust modelling.

### Energy consumption

**Fig. 9:**
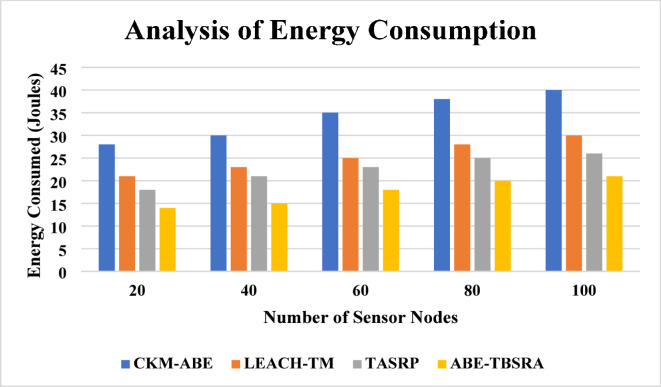
Analysis of energy consumption.

The results of the analysis obtained on the energy consumption metric is shown in Fig. 9. In this work, the proposed ABE-TBSRA algorithm has been evaluated by calculating and comparing the energy consumption of the proposed protocol for WSNs with the three existing protocols namely CKM-ABE^8^, LEACH-TM^19^ and TASRP^20^. The experiments have been carried out with several numbers of sensor nodes such as 20, 40, 60, 80 and 100.

**Fig. 10:**
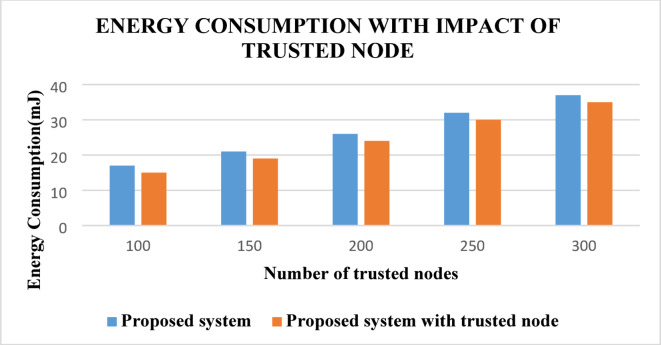
Energy consumption with the impact of trusted nodes.

From this Fig. 9, it is observed that the energy consumption of the sensor nodes is decreased in the network along with the routing process using the proposed ABE-TBSRA than the existing protocols namely CKM-ABE^8^, LEACH-TM^19^ and TASRP^20^. Moreover, the security model developed using ABE with bilinear and Diffie–Hellman and trust management for secure routing in the network enabled the reduction of energy consumption by preventing the attackers from reading the data packets communicated through the network. Figure 10 presents the energy consumption of the proposed system in comparison with the trusted nodes.

### Overall network performance

**Fig. 11:**
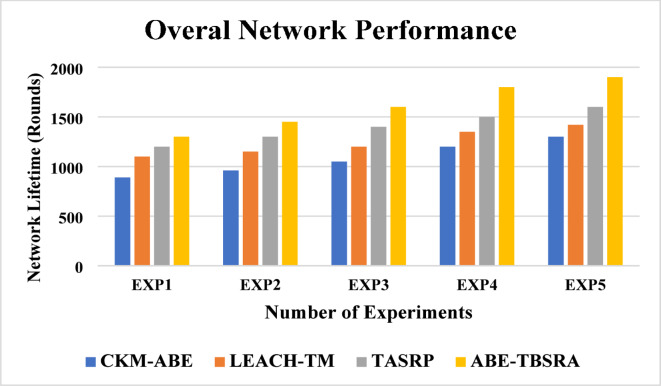
Analysis of overall network performance.

The analysis of overall network performance is shown in Fig. 11. Here, the proposed ABE-TBSRA algorithm has been evaluated by conducting the experiments for analysing the overall performance of the network and by comparing the proposed algorithm with three existing protocols namely CKM-ABE^8^, LEACH-TM^19^ and TASRP^20^.

**Fig. 12:**
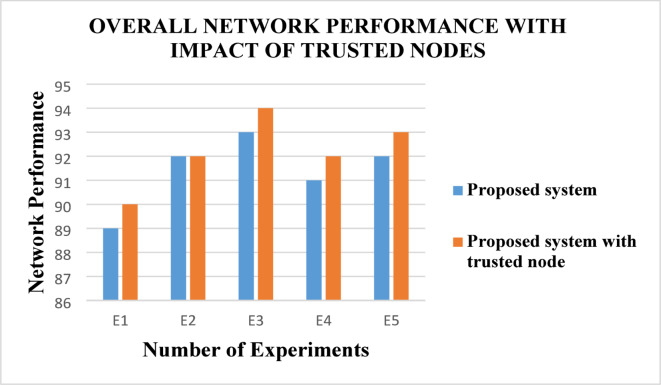
Overall network performance with the impact of trusted nodes.

From this Fig. 11, the overall network performance can be observed in the secure routing algorithm namely ABE-TBSRA developed using ABE and trust method shows the data transmission with high reliability by increasing the network lifetime and it is compared with the other protocols namely CKM-ABE^8^, LEACH-TM^19^ and TASRP^20^. The proposed methods are enhanced due to the authentication, encryption, trust modelling, clustering, CHs, and secure routing process using security methods through the CHs. Figure 12 presents the overall network performance of the proposed system in comparison with the trusted nodes.

## Conclusion

In this paper, a novel proposed algorithm called ABE-TBSRA along with clustering and security techniques to secure the data in an efficient method using WSNs. In this work, we used attribute-based encryption and key management to form the secure routing algorithm that uses clustering and cluster-based routing for reducing the power consumption. Moreover, it provided facilities for CH rotation after each round. A trust management scheme is also present in this work by using the results obtained from the bilinear pairing and Diffie–Hellman scheme along with direct trust, indirect trust and overall trust values. From these experiments, we observed that the security of the routing process is increased when it is compared with other existing secure routing protocols. Future work, in this direction can be the inclusion of an intrusion detection system by exploring federated learning for improving the security. Moreover, block chain based light weight authentication protocols which can be further explored in providing better security with optimized energy consumption for the nodes of WSN.

## Data Availability

All data generated or analyzed during this study are included in this published article.
